# Utilization and Comparative Effectiveness of Caspofungin and Voriconazole Early after Market Approval in the U.S

**DOI:** 10.1371/journal.pone.0083658

**Published:** 2014-01-10

**Authors:** Sibel Ascioglu, K. Arnold Chan

**Affiliations:** Department of Epidemiology, Harvard School of Public Health, Boston, Massachusetts, United States of America; Louisiana State University, United States of America

## Abstract

**Objectives:**

Both caspofungin and voriconazole were initially approved by the FDA with very narrow indications. Our aim was to evaluate the utilization patterns and comparative effectiveness of these agents early after marketing before any labeling change occurred.

**Methods:**

This was a retrospective cohort study utilizing a large healthcare database in the United States. Patients who received at least one dose of systemic antifungal agent between the years 2001 and 2003 were included. Information was available for each hospital-day including underlying conditions, medications, procedures and disease severity scores. Tests for proportions, trend tests and logistic regression were used for evaluation of utilization. Propensity score analysis was used in comparison of mortality.

**Results:**

The study cohort included 381,245 patients with serious underlying conditions. In just two years after marketing, caspofungin and voriconazole use increased to 40% of the total systemic antifungal consumption. However, only 3.4% of caspofungin and 12.5% of voriconazole were used as indicated in labeling. In the propensity score analyses, caspofungin was associated with 7% decrease in mortality (OR: 0.93 95% CI: 0.85–0.98). Voriconazole use was not found to be associated with mortality (OR: 1 . 95% CI: 0.89–1.12)

**Conclusions:**

Caspofungin and voriconazole were mostly used of unapproved indications immediately after their marketing. Although unapproved drug use might be due to a crucial need by clinicians, this may create problems in further antifungal drug development. Our results suggest a survival benefit with caspofungin; however, similar comparative effectiveness studies must be repeated using more recent data.

## Introduction

Amphotericin B deoxycholate (AMB), since its marketing in 1959, has been the mainstay for treatment of the most serious invasive fungal infections (IFIs) [1]. During 1990s, other wide spectrum antifungal agents, such as itraconazole and lipid formulations of amphotericin B (LF-AMB), were introduced. Despite the availability of these agents, potential toxicity limited their use and case fatality rates for IFIs remained high [2], [3]. Two novel antifungal drugs, caspofungin and voriconazole, became available in the U.S. in January 2001 and May 2002, respectively. These two agents were considered by many, as a significant progress in treatment of IFIs, owing to their wide spectrum and lower toxicity [4], [5].

Initially, caspofungin was approved for a single indication; “The treatment of invasive aspergillosis in patients who are refractory to or intolerant of other therapies i.e., amphotericin B, lipid formulations of amphotericin B and/or itraconazole” [6]. Voriconazole received approval for two indications; “Treatment of invasive aspergillosis, and treatment of serious fungal infections caused by *Scedosporium apiospermum* and *Fusarium* spp. in patients intolerant of, or refractory to other therapy” [7]. Traditionally, market approval of antifungal agents has relied on small randomized trials, studies with historical controls or observational data, rather than adequately powered trials with concurrent controls [8]. As a result, most wide-spectrum antifungals i.e. lipid formulations of AMB, itraconazole and caspofungin, were all initially approved for second-line or salvage therapy. It is a well known fact that off-label use occurs frequently in most therapeutic areas which can sometimes be more frequent than those for the approved indications [9]. Although wide spread use of antifungals without supporting evidence has raised concerns for the emergence of resistance and adverse events [10], [11], there is limited information on the efficacy and utilization patterns of systemic antifungals in routine clinical practice [12]–[16.

Our main objective in this study was to evaluate how the marketing of voriconazole and caspofungin changed antifungal utilization in hospitals. Our secondary objective was to determine if caspofungin and voriconazole improved survival, compared to older wide spectrum agents.

## Methods

### Data and study population

Ethics statement: This analysis was carried out with completely de-identified data and in full compliance with the Health Insurance Portability and Accountability Act of 1996 in the U.S.

This was a retrospective cohort study utilizing a large automated healthcare data source in the U.S. We evaluated utilization of caspofungin and voriconazole with regard to approved indications during a period immediately after their marketing and before any labeling change occurred. The study population included patients who received at least one dose of systemic antifungal agent in 507 different hospitals, between January 1, 2001 and December 31, 2003. This retrospective cohort was drawn from the Premier Perspective Database, which is a hospital data warehouse that includes approximately one sixth of all hospitalizations in the US [17]. It is a service-level database providing detailed hospital resource utilization data along with patients' primary and secondary diagnoses in the form of International Classification of Diseases 9^th^ Revision Clinical Modification (ICD-9-CM) and procedure codes. For our analysis, information was available at the level of each hospital-day of a patient and included procedures and medications (drug name, strength, and quantity dispensed). Patient-level information included demographics, principal and secondary procedures, length of stay and severity of illness indicators; all patient refined–diagnosis-related group (APR-DRG) severity- and mortality-score [18], [19]. These scores range from 1 to 4 (minor, moderate, major, extreme) and provided a measure of how ill a patient was relative to other patients in the study population.

### Drug exposure and study outcome

For the mortality and off-label use analyses, exposure was defined as the use of a systemic antifungal agent ≥3 days. Agents which were rarely used (defined as <500 patients in the database) were excluded from the analysis. The diagnosis of a fungal infection was determined by the presence of an ICD-9-CM code in any diagnostic position (admission, primary or secondary discharge diagnoses). In mortality related analyses, older drugs with a wide spectrum of antifungal activity similar to caspofungin and voriconazole (i.e. itraconazole, AMB and LF-AMB) were grouped as “older antifungals”. We excluded patients who had only used oral formulations of itraconazole and voriconazole from the mortality analyses due to the higher likelihood of oral formulations being used for prophylaxis and also due to problems reaching therapeutic levels in the serum. The primary outcome of interest was a discharge status of death according to Universal Billing 92 (UB92) hospital claims form.

### Statistical analysis

For descriptive analyses of antifungal drug utilization, cross tabulations and tests for the comparison of proportions were employed. Factors associated with off-label use of caspofungin and voriconazole were evaluated by multivariable logistic regression.

For the comparison of in-hospital mortality rates between caspofungin or voriconazole and older agents, we used propensity scores (PSs) to control for potential confounders. Propensity scores have become an increasingly popular method to efficiently control large numbers of confounders in database studies [20], [21]. Propensity score is the predicted probability that an individual would have been treated with a particular antifungal agent, based on that individual's observed pretreatment characteristics [22], [23]. The estimated PSs for caspofungin or voriconazole treatment were obtained from two separate logistic regression models, each with a dependent binary variable which was an indicator of the use of caspofungin or voriconazole vs. the use of older antifungals. Covariates in the models were fungal infection diagnoses and pre-treatment variables including comorbidities and disease severity (see Table S1). We used the c-statistic to evaluate the performance of the variables in predicting caspofungin or voriconazole use [24].

For our final analysis, which compared the mortality rates among drugs, we employed two different PS-related methods to control for potential confounders, as each addressed a different research question [21]:

1. One-to-one ‘greedy-match’ on the PSs. We matched each patient in the new agent group (caspofungin or voriconazole users) to a patient in the older-antifungal-user group with the closest PS [23]. This approach creates two populations (i.e. new vs. older antifungal users), which are very similar in terms of confounding factors, therefore allowing comparison of drug effects in these two groups.
2. Standardized-mortality-ratio (SMR) weighted logistic regression model [21]. SMR-weighted analysis uses the value “**1**” for the treated and the propensity odds for the untreated as weights. Thus, it estimates a standardized effect measure, which considers the exposed group as the *standard population* [21], [25]. In other words, this approach transforms the whole study population to a population whose distribution of risk factors is equal to that for new agent treated patients only (i.e. had all of our study cohort subjects been like the caspofungin or voriconazole treated patients).

All analyses were performed using STATA 9.2 (StataCorp., USA).

## Results

Between 2001 and 2003 inclusive 381,245 patients were administered at least one dose of a systemic antifungal drug in the Premier Database. Patients were mostly adults (96%) with severe underlying diseases, multiple comorbidities and prolonged hospital stay (median 11 days, 99^th^ percentile: 94 days). The most common underlying conditions were malignancy (49%), hematopoietic stem cell transplantation (HSCT) (26%) and major surgical operation (29%). Patients were mostly in the major/extreme category (65%) according to the DRG based severity index. Important characteristics of patients and institutions are summarized in Table 1.

**Table 1:** Descriptive characteristics of the study population. Patients who used at least one systemic antifungal agent in the hospital between the years 2001–2003.

Characteristics	Frequency (%) N = 381,245
**No of episodes according to year of discharge**	
2001	117,633 (31)
2002	130,123 (34)
2003	133,489 (35)
**Teaching hospital**	104,104 (27.3)
**Hospital size**	
<250 beds	83,106 (21.8)
250–500 beds	102,734 (42.7)
>500 beds	135,405 (35.5)
**Region**	
Midwest	65,544 (17.2)
Northeast	31,603 (8.3)
South	241,240 (63.3)
West	42,858 (11.2)
**Patients hospitalized more than once**	46,209 (15)
**Age**	
< = 17 yrs	13,402 (3.5)
18–64 yrs	189,802 (49.8)
> = 65 yrs	178,041 (46.7)
**Female**	232,853 (61)
**DRG based severity index**	
Minor	33,010 (8.7)
Moderate	100170 (26.7)
Major	136,749 (35.9)
Extreme	111,270 (29.2)
**Death during hospitalization**	
Expired	47,012 (12.3)
**Median length of stay, days (25^th^–75^th^ percentiles)**	11 (6–20)
**Payor**	
Medicare	202,821 (53.2)
Medicaid	47,774 (12.5)
Managed care	79,794 (20.9)
Other	50,856 (13.3)
**Underlying diseases**	
HIV	20,818 (5.5)
Acute leukemia	11,585 (3.0)
Other hematological malignancies	17,721 (4.7)
Hematopoietic stem cell transplant	99,394 (26.1)
Solid tumor	58,503 (15.3)
Major surgery	86,937 (22.8)
Rheumatoid arthritis	7,051 (1.8)
Systemic lupus erythematosus	3,926 (1.0)

### Utilization and uptake of caspofungin and voriconazole

**Figure 1:**
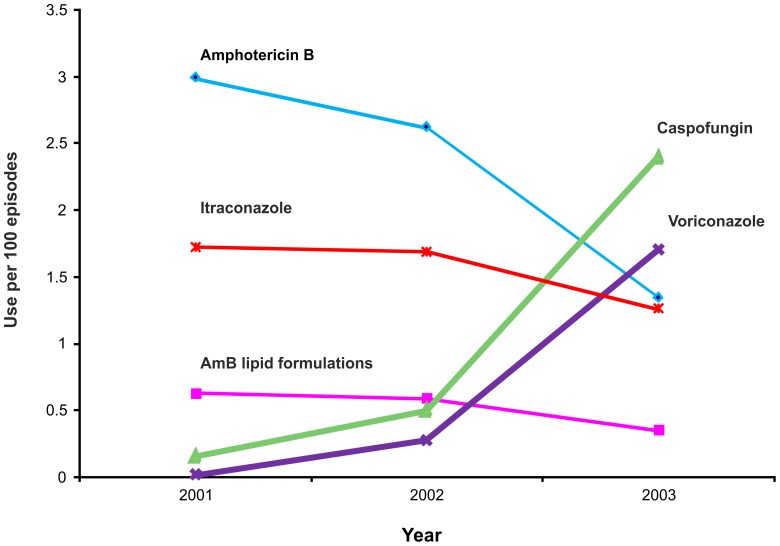
Change in the rates of systemic antifungal use during the study period. The rate of increase for caspofungin was 2.94/100 hospitalization, each year (95% CI: 2.83–3.06) and for voriconazole 3.43/100 hospitalization, each year (95%CI: 3.27–3.60).

During the study period, the most widely used systemic antifungal was fluconazole; 94% of the patients received at least one dose during the study period. When patients who received only fluconazole were excluded from the cohort, the most commonly used systemic agent was AMB (30%) followed by LF-AMB (29.3%) and itraconazole (21%). Between 2001 and 2003, there was a significant increase in the use of two recently approved agents; caspofungin use increased from 2% to 24.5% (p-value for linear trend <0.001) and voriconazole use increased from 3% to 17.4% (p-value for linear trend <0.001) (Table 2), while the use of all other systemic antifungal agents decreased significantly. Patients were almost three times more likely to receive caspofungin and 3.4 times more likely to receive voriconazole each following year (Figure 1). Most of the patients (95%) received only one antifungal agent during a single episode, 4.8% of patients received two agents and 0.2% of patients used three or more.

**Table 2:** Number of hospitalization episodes with at least one dose of systemic antifungal use between 2001 and 2003.

	2001			2002			2003			Total		
	All N = 117,633		W/o Flu^a^ N = 12,507	All N = 130,123		W/o Flu^a^ N = 14,272	All N = 133,489		W/o Flu^a^ N = 16,587	N = 381,245		W/o Flu^a^ N = 43,366
**Fluconazole**	110,699	94.1%	NA	122,332	94.0%	NA	125,101	93.7%	NA	358,132	93.9%	NA
**AMB** b	5025	4.3%	40.2%	5060	3.9%	35.5%	3011	2.3%	18.1%	13096	3.4%	30.2%
**LF-AMB** c	4037	3.4%	32.2%	4519	3.5%	31.7%	4057	3.0%	24.5%	12613	3.2%	29.3%
**Itraconazole**	3718	2.7%	25.4%	3388	2.6%	23.8%	2581	1.9%	15.6%	9147	2.4%	21.1%
**Caspofungin**	245	0.2%	2.0%	880	0.7%	6.2%	4056	3.0%	24.5%	5181	1.4%	11.9%
**Voriconazole**	22	0.0%	0.0%	425	0.3%	3.0%	2882	2.2%	17.4%	3329	0.9%	7.7%

Since fluconazole constitutes a majority of use, a separate column shows use when patients who only used fluconazole are excluded (W/o flu column).

^a^ W/o flu: Patients who used only fluconazole were excluded.

^b^ AMB: Amphotericin B deoxycholate.

^c^ LF-AMB: Lipid formulations of amphotericin B.

### Use according to FDA approved indications

Caspofungin was initially approved as a second-line agent in the treatment of invasive aspergillosis whereas voriconazole was approved for the first-line treatment of aspergillosis. However, patients who had ICD-9-CM codes indicating aspergillosis infection constituted only a minority; 5.2% in caspofungin users and 12.5% in voriconazole users (Table 3). Both caspofungin (58.8%) and voriconazole (64.7%) were mostly given to patients without a specific fungal infection diagnosis. Caspofungin was given as the first-line treatment in 83.5% of the episodes. However, caspofungin was used as approved by the FDA in only 176 patients (3.4%), i.e. in a patient with aspergillosis and after treatment with another agent.

**Table 3:** Use of caspofungin and voriconazole according to FDA approved indications and factors associated with increased unapproved use.

Characteristic	Caspofungin (%) (N = 5181)	Voriconazole (%) (N = 3329)
**Underlying fungal infection**		
*Aspergillus* infection	269 (5.19)	416 (12.50)
*Candida* infection	1794 (34.63)	755 (22.68)
Systemic *Candida* infection	786 (15.2)	232 (6.97)
Other specified infection	38 (0.73)	45 (1.35)
Unspecified mycosis	238 (4.59)	160 (4.81)
No fungal infection diagnosis	3047 (58.81)	2153 (64.67)
**Started as**		
1^st^ line drug	4331 (83.45)	2667 (79.95)
2^nd^ line drug	712 (13.72)	547 (16.4)
3^rd^ drug or later	147 (2.83)	122 (3.66)

Factors associated with off-label use	Odds ratio (95% CI)	Odds ratio (95% CI)
Age		
<17 yrs	1	1
18–64 yrs	4.33 (2.34–8.02)	1.04 (0.36–2.98)
>65 yrs	7.18 (3.63–14.17)	0.78 (0.25–2.47)
Female gender	1.20 (0.94–1.80)	1.24 (0.70–2.23)
Year (From 2001 to 2003)	2.70 (2.14–3.32)	3.80 (1.90–7.56)
APR mortality risk group		
Minor	1	1
Moderate	0.26 (0.06–1.13)	1.02 (0.15–7.12)
Major	0.10 (0.02–0.37)	0.47 (0.07–3.10)
Extreme	0.05 (0.01–0.22)	0.26 (0.04–1.79)
Emergency admission	0.65 (0.43–0.97)	0.45 (0.22–0.91)
Systemic *Candida* infection	3.92 (2.10–7.32)	2.74 (1.09–6.90)
Major surgery	2.58 (1.70–3.92)	0.99 (0.51–1.93)
Sepsis	2.90 (1.96–4.25)	2.86 (1.45–5.64)
Attending physician specialty		
Critical care	0.90 (0.27–2.91)	0.62 (0.10–3.66)
Infectious diseases	0.30 (0.13–0.69)	0.07 (0.01–0.40)
Hematology-Oncology	0.23 (0.13–0.41)	0.32 (0.12–0.86)
Surgeon	0.86 (0.39–1.88)	0.95 (0.28–3.20)
Pulmonology	0.12 (0.06–0.22)	0.10 (0.03–0.30)

In a multivariable logistic regression model, the odds ratio (OR) for off-label use of caspofungin increased significantly each year between 2001 and 2003 (OR: 2.70; 95% confidence interval (CI): 2.14–3.32). Also, older patients (OR: 7.18; 95% CI: 3.63–14.17 for age>65 yrs.), patients who had systemic *Candida* infections (OR: 3.92; 95% CI: 2.10–7.32), patients who underwent major surgery (OR: 2.58; 95% CI: 1.70–3.92) or patients who had sepsis (OR: 2.90; 95% CI: 1.96–4.25) were more likely to receive caspofungin with unapproved indications (Table 3). Patients with higher risk of mortality or emergency admission were less likely to receive unapproved treatment, as were the patients who had a Pulmonologist, Infectious Diseases or Hematology-Oncology specialist as their attending physician. Similar to caspofungin, the OR for off-label use of voriconazole increased significantly each year (OR: 3.80; 95% CI: 1.90–7.56). Systemic *Candida* infection (OR: 2.74; 95% CI: 1.09–6.90) and sepsis (OR: 2.86; 95% CI: 1.45–5.64) were other factors associated with unapproved use. Emergency admission, having an attending physician specialized in Infectious Diseases, Hematology-Oncology or Pulmonology decreased the likelihood of receiving voriconazole with an off-label indication (Table 3).

### Comparison of mortality

In our study cohort, a total of 47,012 patients (12.3%) died during hospitalization (12.5% in 2001, 12.6% in 2002 and 11.9% in 2003). There was a small drop in mortality rate in 2003 (p<0.001), when caspofungin and voriconazole use increased to 40% of the total. In unadjusted analyses, the mortality rate was higher in new agent users; 26.7% in patients who received only caspofungin, 17% in those who received only voriconazole and 19.3% in patients who were given one of the older antifungals. The distribution of clinical characteristics among users of different antifungals agents is summarized in Table S1. There were significant differences, caspofungin users were older (78% over 45 yrs.) and in the major/extreme disease severity category, 76%, compared to 60% of voriconazole users and 62% of older agent users in the major/extreme disease severity category. Caspofungin users were also more likely to be admitted with sepsis or for mechanic ventilation. Voriconazole users were similar to older agent users in terms of distribution of age and severity of illness, but voriconazole was more commonly used in acute leukemia (24.5%), other hematologic malignancies (11%) and HSCT patients (12.8%).

The logistic regression model employed to estimate the PSs for caspofungin versus older anti-fungal agents yielded a c-statistic of 0.92, showing a very good discriminatory power as a predictive model. The crude OR for in-hospital mortality comparing caspofungin users with older antifungal users was 1.48 (95% CI: 1.38–1.58); yet, when we matched on PSs mortality, the OR decreased to values less than 1, showing a protective effect, but the 95% confidence interval included the null value. Intriguingly, an SMR weighted model (which used caspofungin-treated patients as the “standard population”) yielded a statistically significant effect (OR: 0.93; 95% CI: 0.85–0.98) showing 7% better survival among caspofungin users compared to older agent users (Table 4).

**Table 4:** Comparison of the estimated treatment effect of caspofungin on mortality using propensity scores–matched analysis and standardized mortality ratio-weighted analyses.

Model type	No	OR^a^	95% CI^b^
Crude model	35417	1.48	1.38–1.58
Matched on propensity scores	10362	0.98	0.87–1.05
SMR weighted	35417	0.93	0.85–0.98

^a^ OR: Odds ratio;

^b^ CI: Confidence interval.

The logistic regression model employed to estimate the PSs for the use of voriconazole yielded a c-statistic of 0.91, again representing a good discriminatory power. The crude OR for in-hospital mortality among voriconazole users was 0.96 (95% CI: 0.88–1.05); matching on PSs showed a 3% survival advantage but it was not statistically significant (OR: 0.97; 95% CI: 0.86–1.09). The SMR weighted model OR was 1, showing a null effect (Table 5).

**Table 5:** Comparison of the estimated treatment effect of voriconazole on mortality using propensity scores–matched analysis and standardized mortality ratio-weighted analyses.

Model type	No	OR^a^	95% CI^b^
Crude model	33922	0.96	0.88–1.05
Matched on propensity scores	6658	0.97	0.86–1.09
SMR weighted	33922	1.00	0.89–1.12

^a^ OR: Odds ratio;

^b^ CI: Confidence interval.

## Discussion

For this study, we included the period just after caspofungin and voriconazole became available in the US but before any change occurred in the FDA approved indications and publication of updated IDSA guidelines. This allowed us to evaluate the utilization and adherence with the approved indications, in a naturalistic, “real-world” setting. During our study period, there was a 40% decrease in the utilization of older agents and 40% increase in that of caspofungin and voriconazole, indicating that older agents were entirely replaced by newer agents. Our results revealed that 96.6% of caspofungin and 87.5% of voriconazole use was for unapproved indications, which also increased each year during the study period. This level of off-label use may be due to multiple factors [26]. First, antifungal treatment for presumed fungal infections is an established indication in neutropenic cancer patients, but clinical trials did not prove efficacy of voriconazole for this indication and results of the caspofungin study were not yet available at that time [27]. Furthermore, only 35% of these patients who had used caspofungin or voriconazole without any fungal infection diagnosis had a diagnosis of cancer. Second, it may be due to the unmet need by the medical community for less toxic and more effective treatment options in the treatment of IFIs, in other populations such as the surgical or critically ill patients. Our analysis showed that both caspofungin and voriconazole were used in patients with more severe diagnoses such as acute leukemia, HSCT, sepsis and patients with the risk of toxicity, such as renal failure or liver necrosis (Table S1). Yet, this is still a “*therapeutic creep*”, adoption of unproven indications in drug use, mostly due to the tendency to equate “newer” with “better” for medicines or the impact of marketing and promotional efforts [28]. Diffusion of new technologies is seldom smooth or achieved selectively among the population that will benefit the most from it [28], [29] but high level of unapproved use, as in this situation, can have worrying consequences for the future. It can damage the expectations that efficacy and safety of drugs have been fully evaluated and undermine the incentives for manufacturers to perform rigorous studies [30].

Confounding by indication is a major threat to the validity of comparative effectiveness studies in naturalistic settings when non-randomized observational data are used [20]; and therefore needs to be addressed in this study. A doctor's diagnostic and prognostic predictions for a patient will affect the choice of the antifungal agent. Consistently preferring one agent that is believed to be more effective for severely sick patients or for a diagnosis which inherently has a higher mortality (such as invasive aspergillosis) will result in spuriously higher crude mortality rates for a drug, unless the effects of these confounding factors are appropriately addressed [20]. The propensity score method is a very efficient method to control for confounding in large healthcare database studies and its use has dramatically increased since 1983, the first time it was proposed [21]. Adjustments using the estimated PSs efficiently control for a large number of confounders that would otherwise bias the results [21], [25]. We employed two different adjustment methods incorporating propensity scores; matching and SMR weighted analysis which allowed more insight to our data. In the evaluation of caspofungin, increased mortality rate in the crude analysis decreased to a non-significant level in the matched analysis and the SMR weighted analysis showed a statistically significant 7% decrease in mortality compared to patients receiving older agents. The SMR weighing is an indirect standardization method which estimated the treatment effect in a population whose risk factors were the same as caspofungin treated patients in this study.(i.e. had all our study patients been like the caspofungin treated patients of the cohort) [21], [25]. This result is actually consistent with the latest information we have today; in the study cohort, albeit off-label, caspofungin was used either for *Candida* infections or empirically (Table 3). These are the two indications, randomized trials have later shown the efficacy of caspofungin [31], [32] and received FDA approval. Hence, we believe our results show that; if our entire cohort had consisted of patients with *Candida* infections or with the conditions requiring empirical treatment, caspofungin would be a better choice than older antifungal agents.

Propensity score analyses did not show a statistically significantly decrease in mortality rates among voriconazole users compared to older agents. This might be due to the fact that voriconazole has superior efficacy in *Aspergillus* infections [33] which consisted of only 12.5% of its use in our cohort. Likewise, a large trial failed to show equivalence of voriconazole to AMB, in empirical treatment; however, this was the most common situation for voriconazole use in our study [27]. It is also possible that the study period was too early to see a clear survival benefit which may have also been diluted by the use in patients who might not be benefiting most from voriconazole.

We acknowledge the limitations of our study. First, the operational definitions for clinical conditions depended on diagnosis codes, which were not verified against patient medical or laboratory records. Our large sample size prohibited this kind of verification, but our analyses were a comparison both across time and across various different antifungal agents, therefore we do not believe that coding problems would have a differential effect between the different drug exposure groups. Furthermore, if there is a misrepresentation of IFI diagnoses in our data, the error would be on the side of over-reporting, because diagnostic coding is affected by incentives to maximize hospital payments but unfortunately, the coding accuracy of IFIs is unknown [34]. Second, we could evaluate only in-hospital mortality, but patients with severe infections or underlying diagnoses are mostly followed as inpatients; therefore, in-hospital mortality is a big component of all-cause mortality. Finally, although our large database included a severity of disease score with very good predictive value and the use of PSs allowed us to control for several confounders, observational studies related to treatment outcomes always carry a risk of bias due to residual confounding.

## Supporting Information

Table S1 **Demographic and clinical characteristics of patients receiving caspofungin, voriconazole and older antifungal agents.** Numbers are percents of patients unless indicated otherwise.
(DOCX)
