# Supplementary material for: Utilization and Comparative Effectiveness of Caspofungin and Voriconazole Early after Market Approval in the U.S
Source: PLoS One. 2014 Jan 10;9(1):e83658. doi: 10.1371/journal.pone.0083658 (PMC3888390; doi:10.1371/journal.pone.0083658)
Supplement: Table S1 — Demographic and clinical characteristics of patients receiving caspofungin, voriconazole and older antifungal agents. Numbers are percents of patients unless indicated otherwise. (DOCX) [file pone.0083658.s001.docx]

**Table S1**: Demographic and clinical characteristics of patients receiving caspofungin, voriconazole and older antifungal agents. Numbers are percents of patients unless indicated otherwise.

| **Characteristic** | **Treatment** | | |
| --- | --- | --- | --- |
|  | **Caspofungin (N=5181)** | **Voriconazole (N=3329)** | **Older antifungals^a^ (N=34,856)** |
| Age |  |  |  |
| <18 yr | 2.99 | 11.44 | 11.66 |
| 18-44 yr | 18.65 | 21.36 | 22.85 |
| 45-64 yr | 35.11 | 35.60 | 30.63 |
| > 65 yr | 43.25 | 31.60 | 34.86 |
| Female gender | 50.80 | 47.22 | 48.72 |
| Year of admission |  |  |  |
| 2001 | 4.73 | 0 | 34.68 |
| 2002 | 16.99 | 12.77 | 37.18 |
| 2003 | 78.29 | 85.57 | 28.14 |
| Fungal infection diagnosis |  |  |  |
| *Aspergillus* | 5.19 | 12.50 | 6.05 |
| Systemic Candida infections | 15.17 | 6.97 | 8.71 |
| Others | 20.83 | 15.86 | 28.53 |
| No fungal diagnosis | 58.81 | 64.67 | 56.71 |
| Teaching hospital | 43.51 | 55.09 | 37.78 |
| Hospital size |  |  |  |
| <250 beds | 12.39 | 8.92 | 15.26 |
| 250-500 beds | 35.50 | 23.46 | 40.27 |
| > 500 beds | 52.11 | 67.62 | 44.47 |
| Transfer from other hospital | 10.91 | 8.77 | 9.54 |
| APR mortality risk group |  |  |  |
| Minor | 6.62 | 12.20 | 13.57 |
| Moderate | 16.87 | 26.85 | 24.33 |
| Major | 27.99 | 30.04 | 30.96 |
| Extreme | 48.52 | 30.91 | 31.14 |
| Insurance |  |  |  |
| Medicare | 52.02 | 39.05 | 42.90 |
| Medicaid | 11.66 | 12.02 | 17.39 |
| Other | 36.33 | 48.93 | 39.71 |
| Comorbidity |  |  |  |
| HIV | 5.64 | 3.24 | 9.55 |
| Solid tumor | 14.26 | 12.65 | 9.89 |
| Acute leukemia | 10.44 | 24.51 | 11.21 |
| Other hematological malignancies | 7.01 | 11.11 | 5.90 |
| Hematopoietic stem cell transplantation | 3.3 | 12.83 | 27.28 |
| Major surgery | 38.58 | 25.41 | 32.17 |
| Acute renal failure | 34.14 | 22.53 | 20.84 |
| Liver necrosis | 2.80 | 1.56 | 1.52 |
| Admission diagnosis |  |  |  |
| Sepsis | 41.85 | 27.46 | 29.61 |
| Pneumonia | 41.27 | 37.76 | 32.22 |
| Fever | 5.02 | 8.05 | 3.44 |
| Mechanic ventilator support | 40.07 | 24.0 | 29.66 |

**^a^** Itraconazole, amphotericin B and lipid/liposomal formulations of amphotericin B.
